# Frequency of Celiac Disease in Children with Beta Thalassemia major

**Published:** 2014-04-20

**Authors:** N Honar, S Kamali, M Karimi

**Affiliations:** 1 Assistant Professor of pediatric gastroenterologist, Department of gastroenterology, Shiraz University of Medical Sciences, Shiraz, Iran.; 2 Resident of Pediatrics, Department of Pediatrics, Shiraz University of Medical Sciences, Shiraz, Iran; 3 Professor of pediatric hematology oncology, Hematology Research Center, Shiraz University of Medical Sciences, Shiraz, Iran

**Keywords:** Celiac disease, beta-thalassemia, growth failure, frequency

## Abstract

**Background:**

We aimed to investigate the frequency of celiac disease in children with β-thalassemia major (B-TM) in Shiraz, southern Iran.

**Materials and Methods:**

In this study, the prevalence of celiac disease in children with B-TM was evaluated. Children with B-TM were screened for celiac disease by ant-tissue transglutaminase (anti-tTG) IgA antibody, IgA level and anti-tTG IgG. A total of 1500 school healthy children in Shiraz with age/sex matched were selected as control group.

**Results:**

A total of 215 B-TM patients with mean age of 12.7 ± 4.4 years, were included into the study (52.1% was male). None of the patients were positive for anti-tTG IgA. Eight cases were IgA deficient in whom anti-tTG IgG was investigated but none of them were positive for anti-tTG IgG. The finding in control group has a seroprevalence of 2% and biopsy proven disease of 0.6%.

**Conclusion:**

Many patients with thalassemia major have multiple non specific symptom that are not justifiable with underlying disease and might be due to atypical celiac disease. We didn't find any case of celiac disease among more than 200 children with β-thalassemia major in Shiraz, southern Iran. So it seems reasonable to screen only those who have features, even not classical, of celiac disease.

## Introduction

Celiac disease, also known as gluten-sensitive enteropathy or nontropical sprue, is an immune-mediated inflammatory disorder of primarily the small intestine that is triggered by the ingestion of gluten in genetically predisposed individuals. The disorder is more common than previously thought, occurring in 0.5 to 1 percent of the general population in most countries (1). In Iran, studies reported the prevalence from 0.5% in Southern Iran to 0.9% in Northern Iran (2,3).

Only a small proportion of cases of celiac disease are clinically recognized because clinical presentation is not always with classic symptom of malabsorption and having no symptom, called silent celiac disease, and the majority of patients present with atypical form of celiac disease(1,4). Classic celiac disease is presented with chronic diarrhea, weight loss, and abdominal distention that are in about 50% of patients. Indeed, there are several non-classic and also non-gastrointestinal features by which patients with celiac disease may present. These manifestations include iron deficiency with or without anemia of otherwise unknown origin, recurrent abdominal pain misdiagnosed as irritable bowel syndrome, aphthous stomatitis, short stature, liver function test abnormalities such as high aminotransferase levels, chronic fatigue, and reduced bone mineral density (1,4). Also, many children with thalassemia major have several symptoms including vague abdominal pain, constipation, growth failure and other manifestations justified. 

There are some case reports in which growth failure and un-treatable hypothyroidism in children with thalassemia lead to final diagnosis of celiac disease as the underlying cause (5-7). Thalassemia and celiac disease share some clinical features. Growth failure is one of the most common problems in children with thalassemia (8,9). Multi-endocrine dysfunction including thyroid dysfunction, glucose intolerance and diabetes mellitus are common complication seen in patients with thalassemia (10, 11).

All of the mentioned problems are also common in celiac disease. Celiac disease is frequent in 4to33.6% of children with short stature, 2.4% of those with diabetes type 1, and 2to5% of those with hypothyroidism (12, 13). It is highly possible that such problems in children with thalassemia never be considered as celiac disease (14). The clinical presentation of celiac disease makes such an iceberg disease from which we only see the tip, while a majority of patients are un-diagnosed (15). Therefore, there are multiple similar symptoms in thalassemia major and celiac disease and recognition and treatment of celiac disease may improve survival of thalassemia major. There are only few case report about celiac disease in patients with beta thalassemia and this is the first case control study about celiac disease in large group of patients with beta thalassemia.

 These evidences lead us to conduct a case-control study in order to investigate whether the frequency of celiac disease among children with β-thalassemia is higher compared with control group. 

## Materials and Methods

This case-control study was conducted on children with β-thalassemia major (B-TM) referring to thalassemia clinics affiliated to Shiraz University of Medical Sciences, southern IRAN during first 6 month of 2012. A control group from the general population was recruited for comparison, so 1500 school healthy age/sex matched individuals (825 male/675 female; mean age9.5 ±1.3 years) were selected as control group from Shiraz, southern Iran. All the children with β-thalassemia major less than 18 years old were included and there were no specific exclusion criteria other than not willing to participate in the study. The diagnosis of B-TM was based on complete blood count and hemoglobin electrophoresis .All the patients were blood transfusion dependent. The study was approved by the Ethics Committee of Shiraz University of Medical Sciences and informed consent was obtained from parents. 

All demographic data of the patients which were studied include age and gender, anthropometric data such as weight, height, body mass index (BMI) and gastrointestinal symptoms associated with celiac disease such as chronic diarrhea, constipation, abdominal pain, loss of appetite, weight loss, and vomiting. Patients were tested for complete blood count, IgA level, and anti-tissue transglutaminase (tTG) IgA. If the patient were IgA deficient, anti-tTG IgG used to be tested in the patient. Serologic tests were performed with human recombinant enzyme-linked immunosorbant assay (ELISA) method using a commercial available kit; (ORGENTEC Diagnos tiKa GmbH, Mainz, Deutschland). For anti-tTG antibody, the upper limit of the normal range (cut-off value), as determined by the manufacturer was 10 U/mL. In our study, upper endoscopy with Pentax endoscope (EPM- 3300; EG 2940 scope) and duodenal biopsy were performed in patients with abnormal serologic results. Collected data were analyzed using the SPSS version 16.0 (SPSS Inc., Chicago, IL, USA). Data were presented as mean ± SD or number (%).We used chi-square test for comparison. P-value less than 0.05 were considered significant.

## Results

A total of 215 B-TM patients with mean age of 12.7 ± 4.4 years, including 52.1% male were selected into the study. Twenty five of patients were not willing to participate in this study. Demographic data and anthropometric characteristics are presented in Table I. Frequency of celiac disease associated symptoms among patients is presented in Table II. The most common symptom was constipation followed by abdominal pain. Hematology and serology tests’ results are shown in Table III. According to the serologic tests, none of the patients were positive for anti-tTG IgA. Eight cases were IgA deficient in whom anti-tTG IgG was investigated but none of them were positive for anti-tTG IgG. In control group,2% of subjects had positive anti-tTG IgA and the prevalence of biopsy proven celiac disease was 0.6% . According to this study ,the prevalence of serology positive and biopsy proven celiac disease in thalassemia major were not higher than control group, respectively (0.069,0.053).

**Tabel I:** Demographic and anthropometric characteristics in patient with B-thalassemia major

	**Number**	**Percent (%)**			
**Gender, F/M**	103/112	47.9/52.1			
	Min.	Max.	Mean		SD
**Age, year**	1	18	12.7		4.4
**weight, kg**	6	67	35.0		13.6
**Zscore(weight) **	-2.13	2.33	0		1.00
**Height, cm**	70	175	139.3		20.2
**Zscore(Height) **	-3.43	1.75	0		1.00
**BMI, kg/m** ^2^	7.7	36.9	17.2		3.6
**Zscore(BMI) **	-2.62	5.42	0		1.00

**Tabel II:** Frequency of celiac disease associated symptoms in patient with B-thalassemia major

	**Number**	**Percent (%)**
**Chronic diarrhea**	0	0
**Abdominal pain**	11	5.1
**Loss of appetite**	7	3.3
**Weight loss**	6	2.8
**Vomiting**	7	3.3
**Constipation**	16	7.4

**Tabel III:** Blood test and serologic tests in patient with B-thalassemia major

	**Min.**	**Max.**	**Mean**	**SD**
**White blood count**	3200	19500	6435	2197
**Hemoglobin**	5.80	11.20	8.66	0.99
**Mean corpuscular volume**	69	98	79.0	4.9
**IgA level**	0.20	7.44	2.26	1.26
**Anti-tTG IgA**	0.00	3.52	0.92	0.68

## Discussion

The spectrum of clinical manifestation of celiac disease is variable and can present with atypical manifestations. Based on some common features between β-thalassemia major and celiac disease such as growth failure and association with endocrine abnormalities and also based on some available case reports, maybe there is a correlation between two diseases in this study, in which we investigated the frequency of celiac disease among children with β-thalassemia major. We found no case of celiac disease among more than 200 children with β-thalassemia major. The control group in 1500 school children in the same area showed seroprevalence of 2% and biopsy proven disease of 0.6% (2). Therefore, comparing to this studies, our sample size is enough to show that the frequency of celiac disease in children with β-thalassemia of our population is not higher than those in the general population. 

The only reported association between β-thalassemia and celiac disease came from three case reports in the whole literatures. In all of these three cases, there has been a clue by which the clinician has looked for celiac disease. One case suffered from growth failure (6), one from anorexia, arrest of weight gain and low stature (7) and the other from a hypothyroidism not responding to treatments (5). According to these case reports and also findings of our study, it is not worthwhile to routinely screen all children with β-thalassemia for celiac disease, but it still seems reasonable to screen those who have features, even not classical, of celiac disease. 

Although none of the patients in our study have positive serology for celiac disease, we cannot conclude that none of them have celiac disease. Several studies have shown that anti-tTG IgA test has limited sensitivity when there is minimal pathological change in the small intestine (16). Emami and colleagues in Isfahan screened patients with typical and atypical symptoms of celiac disease by serological tests and also performed small intestinal biopsy in those with symptoms highly suggestive of celiac disease even in the absence of positive serology. They had found overall sensitivity and specificity of IgA anti-tTG antibody as 38% and 98%, respectively (16). These results were confirmed in another study by Saneian and colleagues on children in Isfahan. They found limited accuracy of the anti-tTG IgA test with 44.4% sensitivity and 100% specificity(17). Therefore, if the patient has symptoms or features highly suggestive of celiac disease, precise evaluations are warranted for diagnosing or ruling out the disease. In such patient, a genetic test for HLA-DQ2 and HLA-DQ8 would be invaluable because such test has a high negative 

predictive value, which means that the disease is very unlikely to develop in persons who are negative for both HLA-DQ2 and HLA-DQ8 (18).

## Conclusion

In conclusion, there was no association between β-thalassemia major and celiac disease in our study. Based on this result we could not suggest routine screening test for celiac disease in all children with β-thalassemia major. However in children with any symptoms or features suggestive of classic or non classic celiac disease, more work up are recommended.
